# Optimization of appropriate antimicrobial prophylaxis in general surgery: a prospective cohort study

**DOI:** 10.1186/s40001-024-01938-w

**Published:** 2024-06-19

**Authors:** Cansu Zeynep Doğan, Nadir Yalçın, Ömer Cennet, Gökhan Metan, Kutay Demirkan, Kaya Yorgancı

**Affiliations:** 1 Department of Clinical Pharmacy, Faculty of Pharmacy, Hacettepe University, 06230 Ankara, Turkey; 2 Department of General Surgery, Faculty of Medicine, Hacettepe University, 06230 Ankara, Turkey; 3 Department of Infectious Diseases and Clinical Microbiology, Faculty of Medicine, Hacettepe University, 06230 Ankara, Turkey

**Keywords:** Surgical site infection, Antimicrobial prophylaxis, Surveillance, Clinical pharmacist, General surgery

## Abstract

**Background:**

Surgical site infections (SSI) are characterized by infections occurring in the surgical incision site, organ or cavity in the postoperative period. Adherence to surgical antimicrobial prophylaxis (SAP) is paramount in mitigating the occurrence of SSIs. In this study, we aimed to evaluate the appropriateness of SAP use in patients undergoing surgical procedures in the field of general surgery according to the American Society of Health-System Pharmacists (ASHP) guideline and to determine the difference between the pre-training period (pre-TP) and the post-training period (post-TP) organized according to this guideline.

**Methods:**

It is a single-center prospective study conducted in general surgery wards between January 2022 and May 2023, with 404 patients pre-TP and 406 patients post-TP.

**Results:**

Cefazolin emerged as the predominant agent for SAP, favored in 86.8% (703/810) of cases. Appropriate cefazolin dosage increased significantly from 41% (129 patients) in pre-TP to 92.6% (276 patients) in post-TP (*p* < 0.001), along with a rise in adherence to recommended timing of administration from 42.2% (133 patients) to 62.8% (187 patients) (*p* < 0.001). The proportion of patients receiving antibiotics during hospitalization in the ward postoperatively decreased post-TP (21–14.3%; *p* = 0.012), as did antibiotic prescription at discharge (16.8–10.3%; *p* = 0.008). The incidence of SSI showed a slight increase from 9.9% in pre-TP to 13.3% in post-TP (*p* = 0.131).

**Conclusions:**

Routine training sessions for surgeons emerged as crucial strategies to optimize patient care and enhance SAP compliance rates, particularly given the burden of clinical responsibilities faced by surgical teams.

**Supplementary Information:**

The online version contains supplementary material available at 10.1186/s40001-024-01938-w.

## Introduction

Surgical site infections (SSI) denote infections occurring at the site of surgical incision, organ, or cavity after surgical intervention [1]. They impose a substantial burden in terms of morbidity, mortality, and healthcare expenditure [2]. SSIs rank as the most prevalent healthcare-associated infections, particularly in low- and middle-income countries, affecting up to one-third of surgical patients [3].

The Centers for Disease Control and Prevention (CDC) Healthcare-Associated Infections (HAI) prevalence survey documented an estimated 110,800 HAIs linked with inpatient surgeries in 2015. According to the National Healthcare Safety Network (NHSN) 2021 HAI data, there was an approximately 3% rise in the incidence of SSIs across all categories of operative procedures in 2021 compared to the preceding year. Despite advancements in infection prevention and control practices, encompassing enhanced operating room ventilation, sterilization techniques, surgical methodologies, and availability of antimicrobial prophylaxis, SSIs persist as significant contributors to morbidity, extended hospital stays, and mortality. Effective feedback of pertinent data to surgeons has been identified as a pivotal element in strategies to reduce SSI risks. A proficient surveillance program entails adopting epidemiologically sound infection definitions, efficient surveillance techniques, classification of SSI rates based on associated risk factors, and data feedback mechanisms [4]. Procedure-specific, multivariate risk models can furnish more dependable, standardized SSI metrics than traditional SSI rates constrained by the conventional NHSN risk index [5]. Surgical antimicrobial prophylaxis (SAP) denotes the preoperative administration of antimicrobial agents to forestall infectious complications resulting from contamination during surgical procedures [6].

Guidelines grounded on robust studies underscore that appropriate SAP ranks among the efficacious measures in preventing SSIs. For optimal efficacy, delineating the proper indication, selecting an agent covering the anticipated pathogens in wound contamination, and administering adequate bactericidal concentrations throughout the incision’s exposure to bacterial contamination are imperative. Guidelines for SAP are acknowledged as pivotal intervention tools in combating antimicrobial resistance. However, adherence to these guidelines remains suboptimal in numerous regions, leading to unnecessary antibiotic utilization. Heightening awareness regarding the significance of rational antibiotic use and adherence to guidelines stands as crucial interventions advocated for the appropriate use of surgical antimicrobials [2].

Globally, approximately one-sixth of hospital-prescribed antibiotics are designated for SAP. Inappropriate antibiotic prophylaxis arises from misguided prescribing practices, encompassing antibiotic selection, dosage, frequency, and duration. As integral members of antibiotic stewardship teams, pharmacists can exert significant influence in curbing inappropriate SAP use [7, 8].

Preoperative doses should be initiated within 60 min (120 min for fluoroquinolones and vancomycin) before surgical incision. Pharmacokinetic alterations may occur in obese patients, necessitating body weight-based dosage adjustments. In patients with renal and/or hepatic impairment, antimicrobial prophylaxis is administered as a single preoperative dose before surgical incision, thereby obviating the need for dose modifications in this patient cohort. Intraoperative redosing becomes imperative to ensure adequate serum and tissue concentrations of the antimicrobial agent in all patients if the duration of the procedure surpasses two half-lives of the drug, or in cases of excessive intraoperative hemorrhage, or extensive burns. New recommendations advocate for either a single dose or a truncated postoperative antimicrobial course lasting less than 24 h. Postoperative antimicrobial prophylaxis need not be continued due to indwelling drains and intravascular catheters [9].

In this study, our objective was to prospectively evaluate compliance of SAP use among general surgery patients undergoing surgical procedures in accordance with the American Society of Health-System Pharmacists (ASHP) guideline and to enhance adherence to the ASHP guideline by informing physicians about the current situation regarding the incidence of SSI.

## Methods

### Study design

#### General data

**Fig.1:**
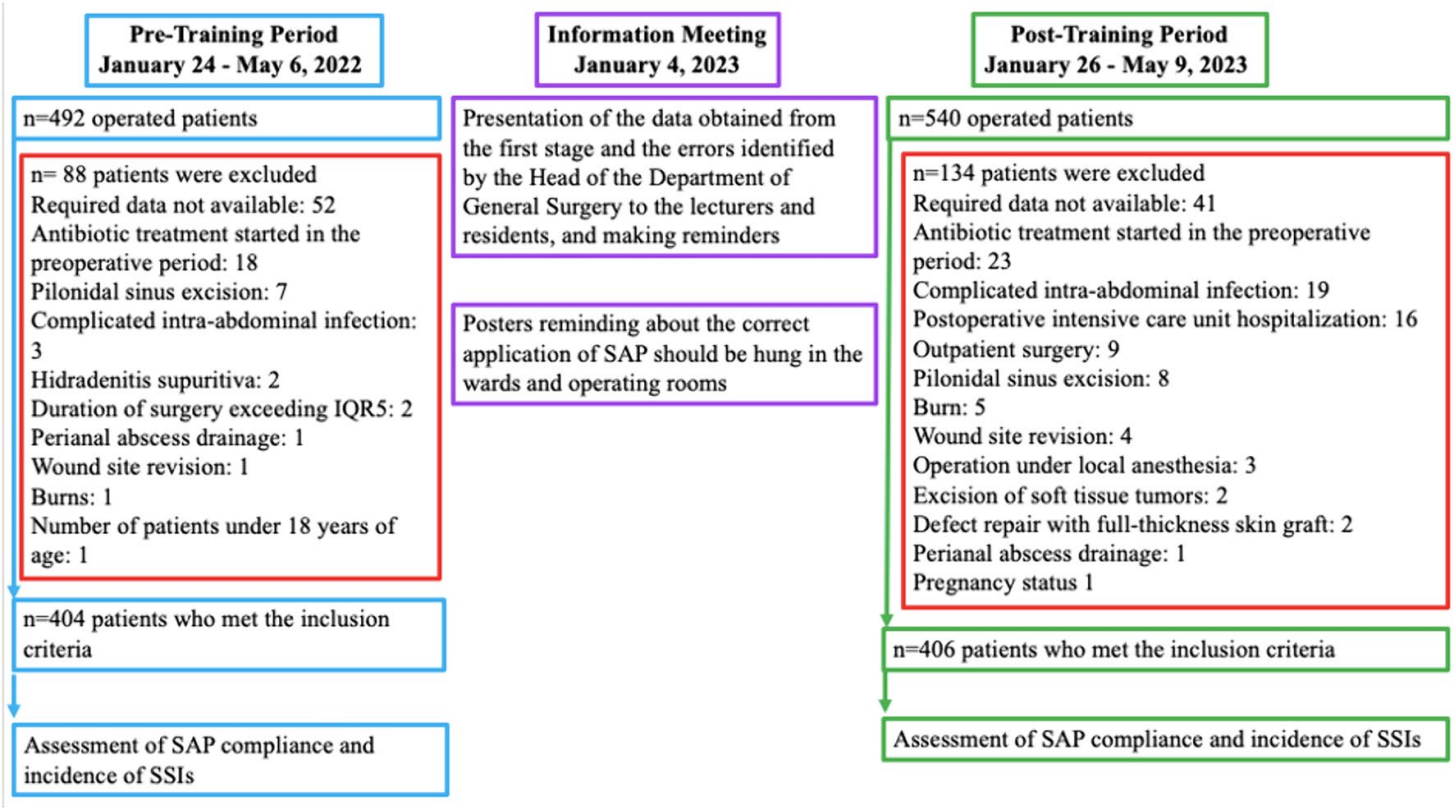
Study flowchart. *IQR* interquartile range, *SAP* surgical antimicrobial prophylaxis, *SSI* surgical site infection

This single-center prospective observational study was conducted in two phases: the pre-training period (pre-TP) spanning from January 24 to May 6, 2022, and the post-training period (post-TP) from January 26 to May 9, 2023, within the general surgery wards of a tertiary care university hospital (Fig. 1).

All individuals aged 18 years and above undergoing emergency or elective surgical procedures with a planned hospitalization duration of at least 24 h in the ward were considered eligible for inclusion. Exclusion criteria encompassed admission to the general surgery intensive care unit or burn unit, individuals with a body mass index (BMI) less than 12 kg/m^2^ or greater than 60 kg/m^2^, patients with an American Society of Anesthesiologist (ASA) score of 6 (a declared brain-dead patient whose organs are being removed for donor purposes), pregnant women, patients with pre-existing infections during surgery, and those receiving antibiotic therapy for any reason before or following surgery. Informed consent was obtained from eligible participants before their inclusion in the study.

Data concerning to demographics, laboratory findings, and the administration of appropriate SAP were meticulously recorded. Compliance rates with SAP and the occurrence of SSIs were retrospectively assessed by a general surgery specialist utilizing patient data.

#### Pre-TP and post-TP data

Findings from the pre-TP were disseminated to faculty members and residents of the general surgery department, besides educational sessions on SAP aligned with guidelines from the ASHP and contemporary scientific literature. After this session, two posters (size of 35 × 50 cm) containing distinct educational content, were prominently displayed in visible areas of the general surgery wards and operating rooms and remained in place throughout the study period. Furthermore, an educational presentation file was circulated via email to faculty members and residents of the general surgery department. Following the training intervention, an assessment was conducted to ascertain whether any improvement was observed during the post-TP. Throughout both phases, patients were contacted by a clinical pharmacist during surveillance periods to assess SSI incidence, and their antibiotic use status was also queried. The ‘Clinical Practice Guidelines for Antimicrobial Prophylaxis in Surgery’, as published by ASHP in 2013, served as the framework for evaluating SAP compliance in our study.

#### Statistical analysis

According to hospital data, the Department of General Surgery conducts approximately 2500–3000 surgeries annually [10]. Additionally, based on existing literature [8, 11], a small effect size was anticipated using Cohen’s *d* statistic between patients included in the study before and after receiving information. Therefore, with an effect size of 0.25, 95% power, and a 5% margin of error, the study planned to include a minimum of 347 patients for both pre-TP and post-TP. Sample size calculation was performed using the *G-power 3.0.10* software.

The results, demographic characteristics, and clinical data of the patients were analyzed using the *SPSS Version 23.0* software. Normality assumptions, a prerequisite for parametric tests, were assessed using the Kolmogorov–Smirnov test and graphical representations. Student’s *T* test was employed for normally distributed numerical data, while the Mann–Whitney *U* test was used for non-normally distributed data. The Chi-square test was utilized to compare ratios. The significance test of the difference between two pairs or the Wilcoxon test was employed to assess changes over time. The relationship between numerical variables was investigated using appropriate correlation tests (Pearson or Spearman). Regression analysis, aimed at quantifying the relationship between a criterion variable and one or more predictor variables, was utilized primarily to determine the nature of the relationship between variables. The McNemar test was employed to ascertain differences between two related groups concerning a dichotomous dependent variable. A significance level of *p* < 0.05 was considered statistically significant.

#### Ethics approval

The study was approved by the Local Ethics Committee (decision no: 2022/17–11).

## Results

### Comparison of demographic characteristics in the pre- and post-training period

A total of 810 patients, 404 in the pre-TP and 406 in the post-TP, were included in the study. Most patients were not allergic to antibiotics in both periods (93.1% and 91.6%;* p* = 0.387). The rate of preferred general anesthesia increased from 94.8% in pre-TP to 99.5% in post-TP (*p* < 0.001). The length of stay in the ward was significantly longer in post-TP than in pre-TP [median (range): 4 (2–61) vs. 3 (2–129) days; *p* = 0.040] (Table 1). No adverse events occurred in any patient who received SAP and/or antibiotics while hospitalized in the ward in the postoperative period.

**Table 1:** Demographic and clinical characteristics of the patients

Variables	Pre-TP (*n* = 404)	Post-TP (*n* = 406)	*p*	Total (*n* = 810)
Age (years), mean (SD)	52.83 (14.34)	53.23 (15.03)	0.711	53.03 (14.68)
Gender (female), *n* (%)	249 (61.6)	258 (63.5)	0.574	507 (62.6)
BMI (kg/m^2^), mean (SD)	27.47 (5.26)	27.57 (4.92)	0.464	27.52 (5.09)
Never smoked cigarette, *n* (%)	240 (59.4)	214 (52.7)	0.155	454 (56.0)
No alcohol use, *n* (%)	357 (88.4)	379 (93.3)	**0.014**	736 (90.9)
Allergic to beta-lactam group antibiotics, *n* (%)	23 (5.7)	23 (5.7)	0.387	46 (5.7)
Diabetes mellitus, *n* (%)	54 (13.4)	59 (14.5)	0.632	113 (14.0)
Malignant neoplasms, *n* (%)	178 (44.1)	196 (48.3)	0.229	374 (46.2)
Regional anesthesia applied, *n* (%)	21 (5.2)	2 (0.5)	** < 0.001**	23 (2.8)
Length of stay (days), median (min–max)	3 (2–129)	4 (2–61)	**0.038**	4 (2–129)
COVID-19 intraoperatively and/or while hospitalized in the ward,* n* (%)	4 (1.0)	2 (0.5)	0.451	6 (0.7)

Boldface font indicates statistically significant variable (p<0.05)

*Pre-TP* pre-training period, *Post-TP* post-training period, *SD* standard deviation, *BMI* body mass index

ASA 2 was the most common ASA score in both periods. The number of patients assigned to ASA 1 decreased in post-TP (10.4% and 2.5%; *p* < 0.001), while the number of patients assigned to ASA 3 increased in post-TP (18.3% and 30.2%;* p* < 0.001).

According to the International Classification of Disease (ICD), patients with at least one comorbidity rate was 91.3% in pre-TP and 95.8% in post-TP (*p* = 0.014). In both periods, the most common diseases were neoplasms, endocrine–nutritional–metabolic diseases, and circulatory system diseases, and no significant difference was observed between the two periods (*p* > 0.05).

The most common types of surgery performed in both phases were herniorrhaphy, breast surgery, gallbladder surgery (Table S1). There was no significant difference between the two periods when the patients were evaluated in terms of the time of hair cleaning and the status of bathing and/or using antiseptic solution within 24 h preoperatively. The rate of emergency surgery was lower in post-TP (6.9% vs. 3.4%;* p* = 0.038) (Tables S2, S3).

Patients in post-TP undergoing elective rectal surgery had a significantly higher preoperative oral antibiotic intake rate compared to pre-TP (66.7% and 95.3% for oral ornidazole; *p* = 0.045, 57.1% and 95.2% for oral cefuroxime; *p* = 0.009). However, no statistically significant change was found on mechanical bowel preparation (MBP) application in rectal and colon surgery (Table S4).

The most preferred prophylactic antibiotic in both periods (810 patients) was cefazolin (703 patients, 86.8%). According to ASHP guidelines, the number of patients who should have received cefazolin was 665 (82.1%) and 613 (92.2%) of these patients received cefazolin. The evaluation of the necessity of giving antibiotics to patients according to ASHP guidelines is shown in Tables 2 and S5. The total number of patients with allergy to beta-lactam group antibiotics was 46 (5.7%).

**Table 2:** Evaluation of the necessity of SAP use according to ASHP guideline (*n* = 810)

Prophylactic antibiotic administration	According to the ASHP, antibiotics are necessary, *n* (%)	According to the ASHP, antibiotics are unnecessary, *n* (%)
Cefazolin was given (n1 = 703)	613 (75.7)	90 (11.1)
Cefazolin was not given (n2 = 107)	52 (6.4)	55 (6.8)
Metronidazole was given (n1 = 78)	62 (7.7)	16 (2.0)
Metronidazole was not given (n2 = 732)	119 (14.7)	613 (75.7)
Ciprofloxacin was given (n1 = 35)	23 (2.8)	12 (1.5)
Ciprofloxacin was not given (n2 = 775)	0	775 (95.7)
Clindamycin was given (n1 = 7)	5 (0.6)	2 (0.2)
Clindamycin was not given (n2 = 803)	1 (0.1)	802 (99.0)
Ampicillin–sulbactam was given (n1 = 5)	2 (0.2)	3 (0.4)
Ampicillin–sulbactam was not given (n2 = 805)	0	805 (99.4)
Ceftriaxone was given (n1 = 2)	0	2 (0.2)
Ceftriaxone was not given (n2 = 808)	0	808 (99.8)
Ampicillin was given (n1 = 2)	1 (0.1)	1 (0.1)
Ampicillin was not given (n2 = 808)	0	808 (99.8)
Amoxicillin–clavulanate was given (n1 = 1)	0	1 (0.1)
Amoxicillin–clavulanate was not given (n2 = 809)	0	809 (99.9)
Levofloxacin was given (n1 = 1)	0	1 (0.1)
Levofloxacin was not given (n2 = 809)	0	809 (99.9)
Vancomycin was given (n1 = 1)	1 (0.1)	0
Vancomycin was not given (n2 = 809)	0	809 (99.9)

*ASHP* American Society of Health-System Pharmacists, *n* Total number of patients, *n1* number of patients given antibiotics, *n2* number of patients not given antibiotics

### Comparison of SAP compliance in the pre- and post-training period

The rate of appropriateness of antibiotic selection according to the ASHP guideline decreased in post-TP compared to pre-TP in patients who received cefazolin, but this decrease was not statistically significant (89.5% vs. 84.9%; *p* = 0.069). There was no significant change between the two periods in the appropriateness of selection of other antibiotics according to the ASHP guidelines (Table 3).

**Table 3:** Evaluation of the appropriateness of antibiotic selection according to ASHP guidelines

Prophylactic antibiotics	Antibiotic selection is appropriate according to ASHP guideline			
	Pre-TP, n1 (%)	Post-TP, n2 (%)	*p*	Total, n3 (%)
Cefazolin (n1 = 352, n2 = 351, n3 = 703)	315 (89.5)	298 (84.9)	0.069	613 (87.2)
Metronidazole (n1 = 38, n2 = 40, n3 = 78)	28 (73.7)	34 (85.0)	0.339	62 (79.5)
Ciprofloxacin (n1 = 20, n2 = 15, n3 = 35)	13 (65.0)	10 (66.7)	> 0.05	23 (65.7)
Ampicillin–sulbactam (n1 = 3, n2 = 2, n3 = 5)	2 (66.7)	0	> 0.05	2 (40.0)
Ceftriaxone (n1 = 2, n2 = 0, n3 = 2)	0	–	–	0
Ampicillin (n1 = 1, n2 = 1, n3 = 2)	1 (100)	0	> 0.05	1 (50.0)
Levofloxacin (n1 = 0, n2 = 1, n3 = 1)	–	0	–	0
Amoxicillin–clavulanate (n1 = 0, n2 = 1, n3 = 1)	–	0	–	0
Clindamycin (n1 = 0, n2 = 7, n3 = 7)	–	0	–	5 (71.4)
Vancomycin (n1 = 0, n2 = 1, n3 = 1)	–	1 (100)	–	1 (100)

*ASHP* American Society of Health-System Pharmacists, *Pre-TP* pre-training period, *Post-TP* post-training period, *n1* number of patients who received antibiotics in the pre-TP, *n2* number of patients who received antibiotics in the post-TP, *n3* total number of patients who received antibiotics

The dose appropriateness rate of patients whose cefazolin selection was appropriate according to the ASHP guideline increased in post-TP compared to pre-TP (41 and 92.6%; *p* < 0.001) (Table 4).

**Table 4:** Evaluation of dose appropriateness rates of patients with appropriate antibiotic selection (according to ASHP guidelines)

Total number of patients with appropriate antibiotic selection according to ASHP guideline	Antibiotic dose appropriate according to ASHP guideline			
	Pre-TP, n1 (%)	Post-TP, n2 (%)	*p*	Total, n3 (%)
Cefazolin (n1 = 315, n2 = 298, n3 = 613)	129 (41.0)	276 (92.6)	** < 0.001**	405 (66.1)
Metronidazole (n1 = 28, n2 = 34, n3 = 62)	28 (100)	33 (97.1)	> 0.05	61 (98.4)
Ciprofloxacin (n1 = 13, n2 = 10, n3 = 23)	1 (7.7)	7 (70.0)	0.006	8 (34.8)
Ampicillin–sulbactam (n1 = 2, n2 = 0, n3 = 2)	2 (100)	–	–	2 (100)
Ampicillin (n1 = 1, n2 = 0, n3 = 1)	1 (100)	–	–	1 (100)
Clindamycin (n1 = 0, n2 = 5, n3 = 5)	–	2 (40.0)	–	2 (40.0)
Vancomycin (n1 = 0, n2 = 1, n3 = 1)	–	0	–	0

Boldface font indicates statistically significant variable (p<0.05)

*ASHP* American Society of Health-System Pharmacists, *Pre-TP*: pre-training period, *Post-TP*: post-training period, *n1*: number of patients who received antibiotics in the pre-TP, *n2*: number of patients who received antibiotics in the post-TP, *n3*: total number of patients who received antibiotics

**Fig. 2:**
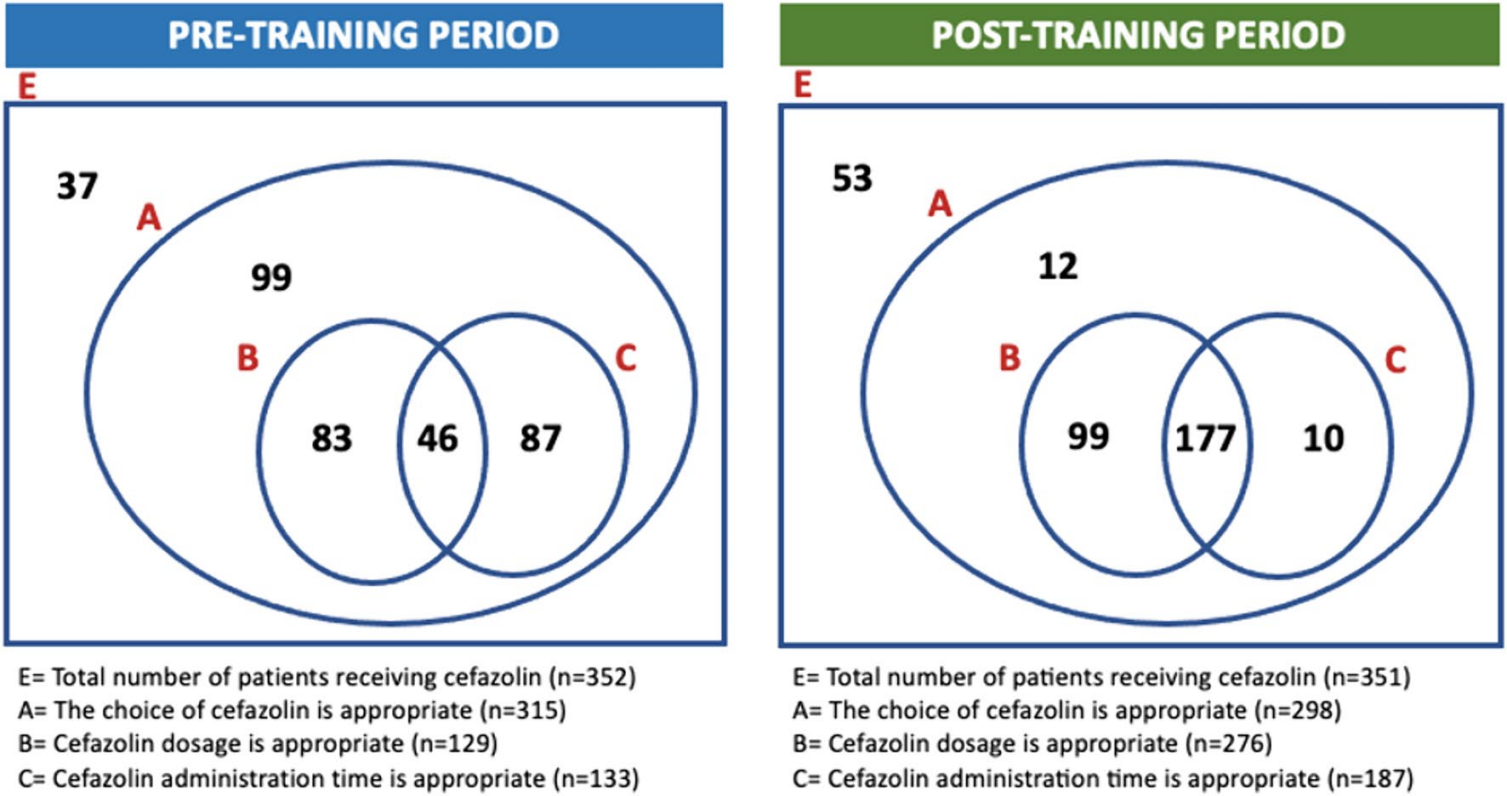
Evaluation of the appropriateness of cefazolin selection, dose and time of administration according to ASHP guidelines

In patients whose cefazolin selection was appropriate according to ASHP guidelines, the appropriateness of the administration time increased after training (42.2 and 62.8%; *p* < 0.001). The appropriateness of the metronidazole administration time also increased after training (35.7 and 73.5%; *p* = 0.006) (Table 5). The comparison of the pre-TP and post-TP of cefazolin, the most preferred antibiotic, is shown in Fig. 2.

**Table 5:** Evaluation of antibiotic administration time appropriateness rates of patients with appropriate antibiotic selection (according to ASHP guideline)

Total number of patients with appropriate antibiotic selection according to ASHP guideline	Patients who could not be evaluated because not specified, *n* (%)		Eligible patients, *n* (%)		*p*	Total patients with appropriate time of administration, *n* (%)
	Pre-TP	Post-TP	Pre-TP	Post-TP		
Cefazolin (n1 = 315, n2 = 298, n3 = 613)	111 (35.2)	111 (37.2)	133 (42.2)	187 (62.8)	** < 0.001**	320 (52.2)
Metronidazole (n1 = 28, n2 = 34, n3 = 62)	14 (50.0)	9 (26.5)	10 (35.7)	25 (73.5)	**0.006**	35 (56.5)
Ciprofloxacin (n1 = 13, n2 = 10, n3 = 23)	5 (38.5)	3 (30.0)	5 (38.5)	7 (70.0)	0.214	12 (52.1)
Ampicillin–sulbactam (n1 = 2, n2 = 0, n3 = 2)	1 (50.0)	–	1 (50.0)	–	–	1 (50.0)
Ampicillin (n = 1, n2 = 0, n3 = 1)	1 (100)	–	0	–	–	0
Clindamycin (n1 = 0, n2 = 5, n3 = 5)	–	1 (20.0)	–	4 (80.0)	–	4 (80.0)
Vancomycin (n1 = 0, n2 = 1, n3 = 1)	–	0	–	1 (100)	–	1 (100)

Boldface font indicates statistically significant variable (p<0.05)

*ASHP* American Society of Health-System Pharmacists, *Pre-TP*: pre-training period, *Post-TP*: post-training period, *n1*: number of patients who received antibiotics in the pre-TP, *n2*: number of patients who received antibiotics in the post-TP, *n3*: total number of patients who received antibiotics

In patients whose antibiotic selection was appropriate according to ASHP guideline, there was no significant difference in the need for intraoperative redosing between pre-TP and post-TP for any antibiotic. The most common reason for antibiotic redosing was the duration of the operation exceeding the half-life of the antibiotic.

When the dose appropriateness of the repeated antibiotics during intraoperative redosing was analyzed according to the ASHP guideline, no significant change occurred in any of the antibiotics (*p* > 0.05). The appropriateness of the time of administration of repeat antibiotics was significantly improved post-TP for cefazolin (14.3% vs. 100%; *p* = 0.005).

The number of patients who developed SSI was 40 (9.9%) in pre-TP and 54 (13.3%) in post-TP (*p* = 0.131). When the 810 patients included in our study were analyzed, the risk of developing SSI increased by 1.9% (*p* = 0.028) with each 1-unit (year) increase in the age of the patients, and the risk of developing SSI increased by 0.7% (*p* < 0.001) with each 10-min increase in the procedure duration. The risk of SSI development was 3.66 times higher (*p* = 0.007) in patients undergoing emergency surgical procedures compared to patients undergoing elective surgery. No statistically significant difference was found between the ASA score increase and the SSI development risk (*p* > 0.05).

## Discussion

In this study, in which the appropriateness of SAP applied to inpatients and operated patients in general surgery wards and the prevalence of SSIs developing in patients were evaluated, cefazolin (86.8%) and metronidazole (9.6%) were the most preferred prophylactic antibiotics in both periods. In a study in which 281 patients were included, the most commonly used antibiotic was the combination of ceftriaxone–metronidazole (45.4%), while the rate of only ceftriaxone use was 33.3% [12]. This is thought to be due to the difference in the approach to the surgical procedures applied between the studies and the difference in drug accessibility.

In our study, 703 of 810 patients received cefazolin for SAP. According to the ASHP guideline, the rate of patients with correct antibiotic selection was 87.2%; the rate of patients with correct antibiotic dose was 66.1%; and patients with correct antibiotic administration time was 52.2%. The ratio of patients who met all three criteria was 31.7%. In another study conducted to evaluate the appropriateness of SAP, it was observed that correct antibiotic selection was observed in 64% of patients, correct antibiotic dose in 34%, correct time of administration in 83% [13]. In our study, the rate of correct antibiotic selection for SAP was found to be higher. However, awareness should be raised not only about the correct choice of antibiotic but also about the appropriate dose and timing of antibiotic administration.

In our study, the cefazolin dose compliance rate significantly increased post-TP according to ASHP guidelines (41 and 92.6%). There was a statistically significant improvement in cefazolin and metronidazole administration timing. The accuracy rate at the time of intraoperative re-administration of cefazolin significantly increased from 14.3% in pre-TP to 100% in post-TP. Therefore, it is thought that sharing the information specified in the ASHP guideline by mentioning the deficiencies in the hospital protocol at the training meeting made a great contribution.

In a study evaluating the impact of educational intervention on SAP compliance in surgical specialties in Turkey, the overall compliance rate decreased in post-TP (34.3% vs 28.5%; *p* = 0.59). Long-term antibiotic use in post-TP was significantly higher (*p* = 0.01). There was a significant decrease in the 'antibiotic administration without indication' rate in post-TP (*p* = 0.009). Although improvements were achieved in the indications, selection and dose of SAP in the study, insufficient success was achieved in improving long-term antibiotic use and overall compliance rate. It has been concluded that surgeons’ adherence to existing protocols and guidelines and educational programs will probably provide better outcomes through mandatory measures to ensure appropriate SAP implementation [14].

In another study conducted by clinical pharmacists in a general surgery ward, 64% of 660 antibiotic prescriptions of 614 patients were found to be inappropriate. The most common cause of inappropriate cases was overuse of antibiotics with a rate of 35.29%. It was observed that inappropriate prescriptions were mostly in cases involving the gastrointestinal system (28%) [15]. In our study, the lowest rate of appropriateness of cefazolin selection according to ASHP guidelines was thyroid and/or parathyroid surgery, with 13.6%, followed by breast surgery, with 89.9%. It is recommended that THYR should not receive antibiotics in the ASHP guideline. Since cefazolin was administered to the majority of patients with THYR, inappropriate use rates were high. The reason why the rate of inappropriate antibiotic use in this study was higher than in our study may be due to the lack of antibiotic stewardship programs and local antibiotic policies. It is thought that the main reason underlying the cefazolin prophylaxis given due to THYR, which was the most frequently observed inappropriate use in our study, was not taking the risk of possible SSI development.

In our study, the SSI development rate was 9.9% in pre-TP and 13.3% in post-TP (*p* = 0.131). The total SSI development rate was 11.6%. In a study conducted by clinical pharmacists in general surgery patients, the criteria for identifying SSI were evaluated according to the definition of SSI in the CDC and SAP compliance according to the ASHP 2013 guideline. SAP was correctly applied in only 19.7% of the total 269 patients, and the incidence of SSI was found to be 16.7%. Independent predictors for SSI were found to be ASA 3–4 (*p* < 0.0001) [16]. In a study of 12,539 patients in 66 countries, the incidence of CAE was 9.4% in high-income countries, 14% in middle-income countries and 23.2% in low-income countries. Therefore, it has been stated that measures should be increased and more randomized controlled studies are needed to reduce the risk of preventable complications in low- and middle-income countries by considering World Health Organization recommendations [17].

In our study, a statistically significant correlation was found between the age of the patients, the duration of the operation, the presence of an emergency surgical procedure and the risk of developing SSI. No significant difference was found between ASA score and the risk of developing SSI. In another study involving general surgery patients, the risk of SSI increased in patients who underwent emergency surgery, in patients aged 60 years and older, for every 10 min of prolonged operation time and in patients with ASA 3 (*p* < 0.05) [18]. In another study involving more than 16,000 general surgery patients, the risk of SSI increased in male patients, in patients with an ASA 3 score, in emergency surgery, and with each 30-min increase in the duration of surgery. No significant relationship was found between age and the risk of developing SSI [19]. In our study, it was realized that SAP was generally not applied to patients undergoing emergency operations. This is thought to be because SAP is not considered due to rapid interventions in emergency procedures and/or not having enough antibiotics on hand. As a result, it was inevitable that the increase in the risk of SSI development in patients undergoing emergency surgical procedures was higher than in other studies. In the patients included in our study, an increase in the risk of SSI development was observed with increasing age and/or prolonged operation time, as in other studies. Still, in our study, unlike other studies, the increase in ASA score did not cause a significant increase in the development of SSI, which gives an idea about which of these risk factors should be emphasized more by evaluating the risk factors that may cause SSI development within each institution.

In our study, beta-lactam allergy was detected in 46 patients, and the SAP that was most preferred in this patient group was ciprofloxacin (32 patients, 69.6%). In 5 (10.9%) of these patients, no antibiotic was administered, and in 3 (6.5%) beta-lactam group antibiotics were administered as SAP. None of the 810 patients had any allergic reaction due to SAP administration. SSI developed in 7 (15.2%) of 46 patients with beta-lactam allergy and 87 (11.4%) of 764 patients without reported beta-lactam allergy (*p* = 0.582). In another study, 11% of patients with beta-lactam allergy received SAP. Vancomycin, levofloxacin, aztreonam and clindamycin were the most preferred agents. No allergic reaction developed in any patient [20]. In another study involving more than 3000 patients, beta-lactam allergy was reported in 369 patients (10.3%). The most preferred agents were clindamycin, gentamicin and vancomycin. SSI developed in 27 (7.3%) patients with beta-lactam allergy and 154 (4.8%) patients without beta-lactam allergy (*p* = 0.03) [21]. The reason why the rate of SAP administration to individuals with beta-lactam allergy was higher in our study compared to other studies may be due to the hesitation of surgeons about the development of SSI and the differences between the precautions taken accordingly. This may have caused the SSIs that developed in the beta-lactam allergic groups in other studies to be significantly higher.

### Study limitations

The limitations of our study are that not all faculty members and resident physicians could attend the training meeting and the training messages could not be conveyed in detail due to the insufficient duration of the meeting. The SAP compliance status given to the patients could not be evaluated in the preoperative process and intervention could not be made accordingly. For the evaluation of SSI development, only telephone interviews were made with the patients and passive surveillance method was preferred. We think that we have increased physicians' awareness on SAP application by including clinical pharmacy practices in general surgery services.

## Conclusion

This study, which identified the gap between SAP guidelines and their clinical application, concluded that the most up-to-date sources should be used in SAP application and hospital protocols should be updated accordingly. While the burden of clinical responsibilities of surgeons is considered, in addition to verbal education and written/posted education materials, sharing the current situation regarding incidences of SSI is also beneficial in the improvement of their practices in adherence to SAP guidelines.

### Supplementary Information

Supplementary Material 1: Table S1. Surgical procedures performed and their distribution. Table S2. Preoperative hair removal status. Table S3. Surgical and preoperative bath/solution use according to the timing of the patients. Table S4. Evaluation of preoperative Mechanical bowel preparation and oral antibiotic use in patients undergoing elective colorectal surgery. Table S5. Evaluation of the necessity of SAP use according to ASHP in patients with allergy to beta-lactam group antibiotics (n=46).

## Data Availability

The data presented in this study are available on request from the corresponding author. The data are not publicly available due to restrictions privacy and ethical.
