# Supplementary material for: Optimization of appropriate antimicrobial prophylaxis in general surgery: a prospective cohort study
Source: Eur J Med Res. 2024 Jun 19;29:340. doi: 10.1186/s40001-024-01938-w (PMC11186073; doi:10.1186/s40001-024-01938-w)
Supplement: Supplementary file 1 — Supplementary Material 1: Table S1. Surgical procedures performed and their distribution. Table S2. Preoperative hair removal status. Table S3. Surgical and preoperative bath/solution use according to the timing of the patients. Table S4. Evaluation of preoperative Mechanical bowel preparation and oral antibiotic use in patients undergoing elective colorectal surgery. Table S5. Evaluation of the necessity of SAP use according to ASHP in patients with allergy to beta-lactam group antibiotics (n=46). [file 40001_2024_1938_MOESM1_ESM.docx]

**SUPPLEMENTS**

**Table S1.** Surgical procedures performed and their distribution

|  | **Pre-TP (n=404)**  **n (%)** | **Post-TP (n=406)**  **n (%)** | **p** | **Total (n=810)**  **n (%)** |
| --- | --- | --- | --- | --- |
| HER | 87 (21.5) | 77 (19.0) | 0.363 | 164 (20.2) |
| BRST | 78 (19.3) | 74 (18.2) | 0.694 | 152 (18.8) |
| CHOL | 62 (15.3) | 78 (19.2) | 0.146 | 140 (17.3) |
| THYR | 39 (9.7) | 59 (14.5) | **0.033** | 98 (12.1) |
| REC | 47 (11.6) | 47 (11.6) | 0.980 | 94 (11.6) |
| SB | 39 (9.7) | 23 (5.7) | **0.045** | 62 (7.7) |
| BILI | 15 (3.7) | 36 (8.9) | **0.004** | 51 (6.3) |
| COLO | 23 (5.7) | 22 (5.4) | 0.986 | 45 (5.6) |
| GAST | 22 (5.4) | 13 (3.2) | 0.162 | 35 (4.3) |
| XLAP | 21 (5.2) | 11 (2.7) | 0.101 | 32 (4.0) |
| APPY | 21 (5.2) | 10 (2.5) | 0.065 | 31 (3.8) |
| NECK | 9 (2.2) | 9 (2.5) | 1.000 | 18 (2.2) |
| NEPH | 7 (1.7) | 4 (1.0) | 0.383 | 11 (1.4) |
| SPLE | 5 (1.2) | 3 (0.7) | 0.505 | 8 (1.0) |
| HYST | 1 (0.2) | 1 (0.2) | 1.000 | 2 (0.2) |
| OVRY | 1 (0.2) | 1 (0.2) | 1.000 | 2 (0.2) |
| PRST | 1 (0.2) | 0 | 0.499 | 1 (0.1) |

Pre-TP: Pre-training period, Post-TP: Post-training period, APPY: Appendix surgery, BILI: Bile duct, liver or pancreatic surgery, BRST: Breast surgery, CHOL: Gallbladder surgery, COLO: Colon surgery, GAST: Gastric surgery, HER: Herniorrhaphy, HYST: Abdominal hysterectomy, NECK: Neck surgery, NEPH: Kidney surgery, OVRY: Ovarian surgery, PRST: Prostate surgery, REC: Rectal surgery, SB: Small bowel surgery, SPLE: Spleen surgery, THYR: Thyroid and/or parathyroid surgery, XLAP: Exploratory laparotomy

**Table S2.** Preoperative hair removal status

|  | **Pre-TP (n=404)**  **n (%)** | **Post-TP (n=406)**  **n (%)** | **p** | **Total (n=810)**  **n (%)** |
| --- | --- | --- | --- | --- |
| No bristle cleaning | 279 (69.1) | 263 (64.8) | 0.195 | 542 (66.9) |
| Hair removal with razor blade | 67 (16.6) | 80 (19.7) |  | 147 (18.1) |
| Performed with a shaver in the operating room | 32 (7.9) | 37 (9.1) |  | 69 (8.5) |
| Hair removal with wax or gloves | 10 (2.5) | 11 (2.7) |  | 21 (2.6) |
| Hair was cleaned with a shaving machine | 9 (2.2) | 8 (2.0) |  | 17 (2.1) |
| Depilatory cream/ Artemisia absinthium powder used | 6 (1.5) | 5 (1.2) |  | 11 (1.3) |
| Hair removal was performed with epilation device | 1 (0.2) | 2 (0.5) |  | 3 (0.4) |
| **Time for hair cleaning** | **Pre-TP (n=125)**  **n (%)** | **Post-TP (n=143)**  **n (%)** | **p** | **Total (n=268)**  **n (%)** |
| Days before surgery | 91 (72.8) | 101 (70.6) | 0.842 | 192 (71.6) |
| Operating room | 31 (24.8) | 37 (25.9) |  | 68 (25.4) |
| Day of surgery | 3 (2.4) | 5 (3.5) |  | 8 (3.0) |

Pre-TP: Pre-training period, Post-TP: Post-training period

**Table S3.** Surgical and preoperative bath/solution use according to the timing of the patients

|  | **Pre-TP (n=404)**  **n (%)** | **Post-TP (n=406)**  **n (%)** | **p** | **Total (n=810)**  **n (%)** |
| --- | --- | --- | --- | --- |
| Elective | 376 (93.1) | 392 (96.6) | **0.038** | 768 (94.8) |
| Emergency | 28 (6.9) | 14 (3.4) |  | 42 (5.2) |
| APPY* | 20 (5.0) | 7 (1.7) | 0.306 | 27 (3.3) |
| HER* | 5 (1.2) | 5 (1.2) | 0.370 | 10 (1.2) |
| XLAP* | 3 (0.7) | 3 (0.7) | 0.383 | 6 (0.7) |
| CHOL* | 3 (0.7) | 0 | 0.539 | 3 (0.4) |
| **Preoperative bath/solution usage status of elective cases** | **Pre-TP (n=376)**  **n (%)** | **Post-TP (n=392)**  **n (%)** | **p** | **Total (n=768)**  **n (%)** |
| Took a bath | 230 (61.2) | 245 (62.5) | 0.705 | 475 (61.8) |
| Used antiseptic solution | 290 (77.1) | 285 (72.7) | 0.158 | 579 (74.9) |

Pre-TP: Pre-training period, Post-TP: Post-training period, APPY: Appendix surgery, CHOL: Gallbladder surgery, HER: Herniorrhaphy, XLAP: Exploratory laparotomy

* Procedure codes for emergency cases (some patients received more than one NHSN operation code).

**Table S4.** Evaluation of preoperative Mechanical bowel preparation and oral antibiotic use in patients undergoing elective colorectal surgery

|  | **Preop MBP was performed, n (%)** | | **p** | **Preop oral ornidazole was given, n (%)** | | **p** | **Preop oral cefuroxime was given, n (%)** | | **p** |
| --- | --- | --- | --- | --- | --- | --- | --- | --- | --- |
| Type of rectal surgery | Pre-TP | Post-TP |  | Pre-TP | Post-TP |  | Pre-TP | Post-TP |  |
| Colorectal n1=21, n2=21 | 20 (95.2) | 21 (100) | >0.05 | 14 (66.7) | 20 (95.3) | **0.045** | 12 (57.1) | 20 (95.2) | **0.009** |
| Hemorrhoidectomy n1=8, n2=10 | 7 (87.5) | 9 (90.0) | >0.05 | 0 | 0 |  | 0 | 0 |  |
| Anal fistula n1=13, n2=8 | 10 (76.9) | 8 (100) | 0.257 | 0 | 0 |  | 0 | 0 |  |
| Anal fissure n1=4, n2=6 | 3 (75.0) | 6 (100) | 0.4 | 0 | 0 |  | 0 | 0 |  |
| Colon surgery n1=23, n2=22 | 22 (95.7) | 19 (86.4) | 0.346 | 14 (60.9) | 19 (86.4) | 0.091 | 13 (56.5) | 19 (86.4) | **0.047** |

Pre-TP: Pre-training period, Post-TP: Post-training period, MBP: Mechanical bowel preparation, n1: number of patients pre-TP, n2: number of patients post-TP,

Note: Both rectal surgery and colon surgery procedures were performed under elective conditions in both periods.

**Table S5**. Evaluation of the necessity of SAP use according to ASHP in patients with allergy to beta-lactam group antibiotics (n=46)

| **Prophylactic antibiotic administration** | **It was needed and given, n (%)** | **It was needed, but it wasn't given, n (%)** | **It wasn't need and it wasn't given, n (%)** | **It wasn't need, but it was given,**  **n (%)** |
| --- | --- | --- | --- | --- |
| Clindamycin or vancomycin | 4 (8.7) | 13 (28.3) | 29 (63.0) | 0 |
| Clindamycin or metronidazole | 3 (6.5) | 6 (13.0) | 37 (80.4) | 0 |
| Clindamycin or vancomycin or metronidazole | 1 (2.2) | 14 (30.4) | 31 (67.4) | 0 |
| Aminoglycoside or fluoroquinolone | 23 (50.0) | 3 (6.5) | 10 (21.7) | 10 (21.7) |
